# The GLVC scoring system: a single-center model for predicting survival and hospitalization in patients with heart failure

**DOI:** 10.1007/s11845-023-03343-4

**Published:** 2023-04-12

**Authors:** Anna Chuda-Wietczak, Agata Sakowicz, Agnieszka Tycinska, Ibadete Bytyci, Agata Bielecka-Dabrowa

**Affiliations:** 1 Heart Failure Unit, Department of Cardiology and Congenital Diseases of Adults, Polish Mother’s Memorial Hospital Research Institute, Rzgowska 281/289, Lodz, 93-338 Poland; 2 Department of Preventive Cardiology and Lipidology, Chair of Nephrology and Hypertension, Medical University of Lodz, Zeromskiego 113, Lodz, 90-549 Poland; 3 Department of Medical Biotechnology, Medical University of Lodz, Lodz, Poland; 4 Department of Cardiology, Medical University of Bialystok, Bialystok, Poland; 5 Clinic of Cardiology, University Clinical Centre of Kosovo, Prishtina, Kosovo

**Keywords:** Global longitudinal strain for left ventricular, Heart failure, High sensitive CRP, Left ventricular diastolic diameter, Oxygen pulse, Prognosis

## Abstract

**Background:**

Heart failure (HF) is the only cardiovascular disease with an ever-increasing incidence.

**Aims:**

The aim of this study was to assess the predictors of adverse clinical events (CE) and the creation and evaluation of the prognostic value of a novel personalized scoring system in patients with HF.

**Methods:**

The study included 113 HF patients (median age 64 years (IQR 58–69); 57.52% male). The new novel prognostic score named GLVC (G, global longitudinal peak strain (GLPS); L, left ventricular diastolic diameter (LVDD); V, oxygen pulse (VO_2_/HR); and C, high sensitivity C-reactive protein (hs-CRP)) was created. The Kaplan–Meier method and log-rank test were used to compare the CE.

**Results:**

Results from final analyses showed that low GLPS (< 13.9%, OR = 2.66, 95% CI = 1.01–4.30,* p* = 0.002), high LVDD (> 56 mm, OR = 2.37, 95% CI = 1.01–5.55, *p* = 0.045), low oxygen pulse (< 10, OR = 2.8, 95% CI = 1.17–6.70, *p* = 0.019), and high hs-CRP (> 2.38 µg/ml, OR = 2.93, 95% CI = 1.31–6.54, *p* = 0.007) were independent prognostic factors for adverse CE in HF population. All the patients were stratified into a low-risk or high-risk group according to a novel “GLVC” scoring system. The Kaplan–Meier analyses demonstrated that patients in the high-risk group were more predisposed to having higher adverse clinical events compared to patients in the low-risk group.

**Conclusions:**

A novel and comprehensive personalized “GLVC” scoring system is an easily available and effective tool for predicting the adverse outcomes in HF.

**Graphical abstract:**

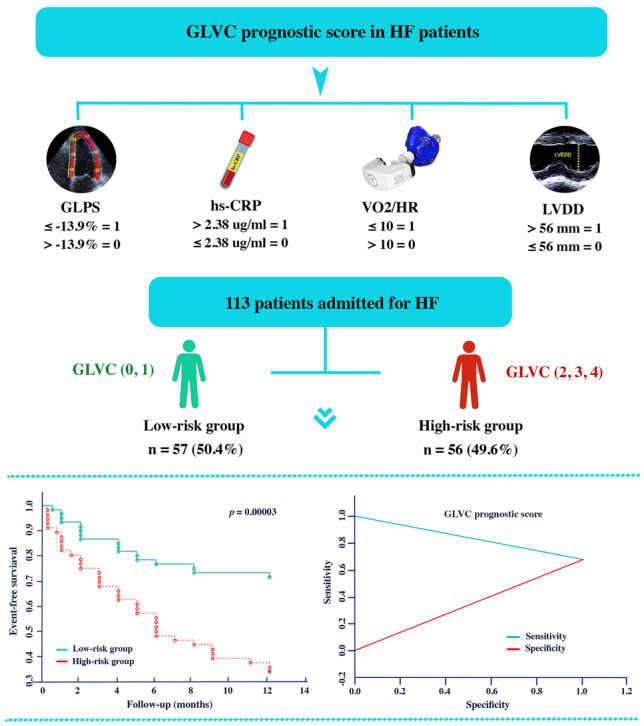

## Introduction

Heart failure (HF) is a common health problem, affecting about 1–2% of adults in developed countries, while in people > 70 years of age, this proportion is > 10% [1–8]. HF is one of the leading causes of death and disability in the world and is characterized by an ever-increasing incidence [1]. It is a significant and growing health and economic problem also in Poland [3, 5, 7, 9–11] associated with high mortality rates (approximately 12–15% annually among patients with moderate stable HF), and nearly half of them die within 4 years of receiving diagnosis [1–8]. The high costs of caring for a patient with HF mostly result from repeated hospitalizations [3–7]. The main challenge in the treatment of HF is the availability of reliable prognostic models that would enable patients and physicians to develop realistic prognosis expectations and to select the appropriate therapy and monitoring methods [12]. Assessment of prognosis plays a special role in patients qualified for treatment with implantable devices or for surgical treatment (including heart transplantation). Prognosis also plays an important role in planning terminal palliative care with the patient and his family [12]. Identifying factors that contribute to a poor prognosis can help develop new, more effective treatment regimens.

There are many variables that inform about prognosis. The research carried out as part of this study will identify independent variables associated with poor prognosis of patients hospitalized for HF.

## Objectives

The aim of the study was to identify independent variables associated with an unfavorable prognosis of patients hospitalized for heart failure based on an available in literature-specific predictive models and create a new prognostic personalized scoring system for the better predictive value for patients with HF.

## Material and methods

### Subject

This is a preliminary study based on a small group. The study included 113 HF patients (median age 64 years (IQR 58–69); 57.52% male).

According to the 2021 ESC Guidelines for the Diagnosis and Treatment of Acute and Chronic Heart Failure [8], HF with preserved ejection fraction (HFpEF) was defined by a left ventricular ejection fraction (LVEF) ≥ 50%. HF with reduced ejection fraction (HFrEF) was defined as LVEF ≤ 40%, and HF with mildly reduced ejection fraction (HFmrEF) if LVEF was 40–49% [8].

Patients were divided into 3 subgroups:

1. HFrEF patients with LVEF <  = 40% [8]
2. HFmrEF patients if LVEF 41–49% [8]
3. HFpEF patients if LVEF ≥ 50% [8]

### Eligibility criteria

The study’s inclusion and exclusion criteria are defined in Table 1.

**Table 1:** Inclusion and exclusion criteria summary

**Inclusion criteria**	**Exclusion criteria**
1. Age equal to or older than 18 years	1. Advanced liver failure (class B and C according to Child–Pugh score) [14]
2. HF (ischemic and non-ischemic) diagnosed according to the 2021 ESC guidelines on HF [8], with HF class I, II, or III according to the NYHA classification [13]	2. Advanced chronic kidney disease (stages G4 and G5 according to the 2012 KDIGO classification [15]
3. Current HF hospitalization	3. Cerebrovascular accident (TIA/ stroke/intracerebral hemorrhage) within 3 months prior to hospitalization
4. LVEF documented in echocardiography during the current hospitalization	4. Current pregnancy or lactation
	5. Alcohol and drug abuse
	6. Active autoimmune disease
	7. Surgery or a serious injury within 1 month prior to hospitalization
	8. Other important medical conditions that could have shortened the survival time during the study
	9. Impaired cognitive status that compromises the understanding of the steps and completion of the study

*HF* heart failure, *ESC* European Society of Cardiology, *NYHA* New York Heart Association, *LVEF* left ventricle ejection fraction, *KDIGO* the Kidney Disease Improving Global Outcomes, *TIA* transient ischemic attack

### Protocol

Every patient underwent a clinical assessment on admission. The following data were included in this analysis: baseline demographics, medical history, medication data, assessment of the severity of HF symptoms according to the NYHA classification [13], and physical examination.

In all patients, the following diagnostic procedures were also performed during hospitalization: selected laboratory tests and concentration of selected biomarkers, Kansas City Cardiomyopathy Questionnaire (KCCQ) score, bioelectrical impedance body mass analysis, Doppler echocardiography, electrocardiogram (ECG), cardiopulmonary exercise test (CPET), and 6-min walk test (6MWT).

One year after discharge, a telephone interview was conducted for the following events: death, cardiovascular adverse events (myocardial infarction, acute coronary syndrome, stroke), and hospitalization for HF. The quality of life according to the KCCQ questionnaire was also re-assessed during the telephone interview.

## Composite endpoint:

- Another hospitalization for HF
- Hospitalization for cardiovascular reasons (myocardial infarction, acute coronary syndrome, stroke)
- Death from any cause

### Measurements

#### Doppler echocardiography

For the clinical and diagnostic stratification, a 2D echocardiogram was performed in all of the 113 patients using a Vivid E95 system (GE Healthcare, Chicago, USA) with a 1.4–5.2 MHz matrix transducer and tissue Doppler imaging software. Body surface area (BSA) was obtained using DuBois and Dubois’ formula [16]. The echocardiographic measurements were performed according to the guidelines of the American Society of Echocardiography (ASE) and the European Association of Echocardiography (EAE) [17, 18]. An echocardiographic assessment was performed on all patients including M-mode and two-dimensional (2D) images. The following echocardiographic parameters were included in this analysis: LVEF, left ventricular end-diastolic diameter (LVDD), left ventricular end-systolic diameter (LVESD), left ventricular end-diastolic volume (LVEDV), left ventricular end-systolic volume (LVESV), left atrial volume index (LAVI), tricuspid annular plane systolic excursion (TAPSE), right ventricular systolic pressure (RVSP), and inferior vena cava (IVC) size. LVEF was calculated using Simpson’s method [17, 18]. Manual tracings of left ventricular endocardial borders were obtained at end-diastole and end-systole in the apical views, and respective volumes were derived using the modified biplane Simpson rule or the Teichholz method, depending on image quality. Left atrial volume was obtained using the biplane area-length method from apical views and indexed to body surface area. TAPSE was averaged over 5 cardiac cycles. RVSP was acquired as a measure of pulmonary artery (PA) pressure. RVSP was estimated based on the average peak gradient of tricuspid regurgitant jets from 5 cycles and adding the estimated RA pressure based on the inferior vena caval diameter and collapse. Routine measurements in size of the IVC and collapsibility with respiration were measured using 2D or M-mode assessment of IVC [17, 18]. Two-dimensional speckle tracking echocardiography was performed in the 2-chamber, 3-chamber, and 4-chamber apical views. The endocardial border was traced by an automated function that defined a region of interest (ROI) at the end-systole. The investigator visually assessed the detected ROI and, if necessary, manually modified the ROI to ensure correct tracking of the speckles. Global longitudinal peak strain (GLPS) was calculated as an average peak strain from the 3 apical projections, and the ROI was set to cover the entire LV. If speckle tracking could not be obtained from a chamber view, GLS was averaged from the remaining chamber views [17, 18].

#### Electrocardiographic data

Standard 12-lead electrocardiograms were recorded using MAC 5500 machines (GE Medical Systems, Milwaukee, USA). Every ECG was recorded on admission. With the initial visual inspection, recordings with inadequate quality were excluded. The MAC 5500 machines measured the QRS duration from the beginning of the Q wave to the end of the S wave in all 12 leads. The MAC 5500 machines measured the QT interval from the onset of the QRS complex to the end of the T wave in all 12 leads. The averaged QT interval was then corrected for heart rate using the Bazett formula (QTc = QT/√RR).

#### Body mass analysis

Bioelectrical impedance analysis (BIA) was performed with the body composition analyzer MC-780MA S (Tanita, Amsterdam, Netherlands), which performs 15 impedance measurements per patient, by measuring the conductance of electrical current across five body segments (legs, arms, and whole body) at three frequencies each (5, 50, and 250 kHz) [19]. The following parameters were included in this analysis: weight, BMI, fat %, fat mass (FM), fat-free mass (FFM), total body water (H2O-TBW), extracellular water (ECW), intracellular water (ICW), extracellular water ratio normalized for total body water (ECW/TBW), and metabolic age (MA).

#### Cardiopulmonary exercise testing

Symptom-limited CPET was performed on an electromagnetically braked upright cycle ergometer Bike M (CORTEX Biophysik GmbH, Leipzig, Germany) with a metabolic gas analyzer METALYZER 3B (CORTEX Biophysik GmbH, Leipzig, Germany) using The MetaSoft Studio application software of CORTEX systems. The system was calibrated with a standard gas mixture of known concentrations before each test. The breath gas analyzer was internally calibrated directly prior to each measurement. The volume of gases and the flow sensor were also calibrated directly prior to the test, validating the calibration twice a year.

After 5 min of rest on the cycle ergometer, exercise commenced at 50 W; then, the work rate was increased by 25 W every 3 min. During CPET, blood pressure was measured by a conventional sphygmomanometer Exacta (Rudolf Riester GmbH, Jungingen, Germany) every 3 min. HR and standard 12-lead electrocardiogram (ECG) were monitored using an exercise electrocardiogram Meta control 3000 (CORTEX Biophysik GmbH, Leipzig, Germany) which connects the CORTEX METALYZER 3B. The criteria for discontinuation of CPET were as follows:

- Maximum heart rate (HR) of 220 beats per minute minus years of age.
- Drop in HR during exercise.
- Drop in systolic blood pressure (SBP) of > 10 mmHg from baseline blood pressure despite an increase in workload.
- Signs of poor perfusion (cyanosis or pallor).
- ST segment depression (> 1.0 mm) or elevation (> 1.0 mm).
- Lack of ventilatory reserve.
- Respiratory exchange rate (RER) higher than 1.15.
- Plateau in consumption of oxygen, with an increase of less than 150 ml/min in about 30 s.
- Personal discomfort (maximal fatigue, thoracic pain, severe dizziness, near syncope, excessive dyspnea, subject signals to examiner to end the examination).
- Severe cardiac arrhythmia (progressive atrial or ventricular arrhythmia or block images, newly occurring atrial fibrillation).

Expired gases were continuously measured in all subjects on a breath-by-breath basis. Uptake of oxygen was measured in a breath-by-breath fashion, recording mean values over 30 s.

Before, during, and after the test, we recorded continuously HR, breathing frequency, minute volume, uptake of oxygen, and elimination of carbon dioxide. Blood gas analyses were recorded at rest (Rest; average of 5 min of rest on the cycle ergometer), at anaerobic threshold (AT), at the exercise peak (Peak), and at 90 s after the end of the effort.

The RER was defined as the ratio between carbon dioxide production (VCO_2_) and oxygen production (VO_2_). A peak RER of > 1.10 was considered an indication of excellent subject effort during CPET.

Peak VO_2_ (VO_2_ max) was defined as the mean of values measured within the last 20 s of exercise and expressed as both ml/min and ml/kg/min (VO_2_ max/kg) and as the percentage of predicted peak oxygen consumption (VO_2_%Norm).

AT was determined by gas-exchange criteria as the point of nonlinear increase in ventilation equivalents for oxygen (ventilation starts to increase at a faster rate than VO_2_).

The minute ventilation/carbon dioxide production relation slope (VE/VCO_2_ slope), the slope of the increase in ventilation to the increase in CO_2_ output, was calculated during incremental exercise using least squares linear regression by the computer system METALYZER 3B.

O_2_ pulse (VO_2_/HR) was defined by dividing VO_2_ by HR.

VO_2_%Norm at AT and Peak were calculated according to published age and sex-normalized values [20].

#### 6-min walk test

The walking tests were conducted according to the standardized protocol [21]. Patients were asked to walk as far as possible in 6 min along a 30-m-long corridor. Standardized instructions and encouragement were commonly given during the test. The test was finished when patients were unable to continue the test or when they were desaturated. The distance walked at the end of 6 min (6MWTD) was recorded in meters.

#### Quality of life

The Kansas City Cardiomyopathy Questionnaire was used to assess the quality of life of the patients. It is a validated instrument to assess health status and quality of life among persons with HF. The self-administered questionnaire includes 23 items which quantify the importance of dyspnea, fatigue, and edema for physical, social, and emotional functions. Patients answer the questions as they related to the previous 2 weeks. An overall summary score can be derived from the physical function, symptom (frequency and severity), social function, and quality of life domains. For each domain, the validity, reproducibility, responsiveness, and interpretability have been independently established. Scores are transformed into a range of 0–100, in which higher scores reflect better health status [22].

#### Laboratory analysis

Approximately 10 ml of blood from a peripheral vein was collected into a tube containing potassium ethylenediamine tetra-acetic acid (1 mg/ml) for the determination of selected biomarkers. The samples were centrifuged at 4 °C for 20 min. The plasma was separated and subsequently frozen at − 70 °C until further analysis without undergoing any additional freeze–thaw cycles. Concentrations of biomarkers were measured by dedicated ELISA immunoassays.

### Statistical analysis

Statistical analysis was conducted using statistical packages STATISTICA PL 13.1.

The Shapiro–Wilk test was used to check the normality of the statistical distribution. All variables with normal distribution were expressed as mean and standard deviation (SD). All variables with no normal distribution were expressed as median and interquartile range (IQR, Q25–Q75). Categorical variables were expressed as the number of observations (*N*) and the corresponding percentage (%).

The Mann–Whitney *U* test was used to compare continuous variables with not normal distribution. The two-tailed Student *t*-test was used to determine whether two populations are statistically different from each other in normal distribution. For multiple comparisons, the Kruskal–Wallis with Dunn post hoc test was used. The *χ*^2^ test was used to compare the qualitative data between the groups.

Spearman’s rank correlation coefficient was used to assess the relationship between the variables measured on an ordinal scale. Data used for multivariate analyses were changed to a dichotomous system according to the median. Backward stepwise logistic regression analysis was used to evaluate the impact of the factors on composite primary endpoint occurrence in multivariate designs. The odds ratio (OR) and 95% confidence interval (95% CI) were reported for each factor in multivariate analysis. Some factors identified as potentially significant by univariate analyses, based on literature data and own clinical experience, were entered into a multivariate analysis to determine the significant independent prognostic factors.

The factors identified by the multivariate analysis were further used to establish a novel prognostic score. The optimal cut-off levels of continuous variables were expressed as median for the whole population.

The quality of individual models and the usefulness of subsequent markers was assessed using ROC curves analysis (with the indication of the sensitivity, specificity, positive and negative predictive ability). The area under the curve (AUC) of receiver operating characteristic (ROC) was used to identify the predictive accuracy of the new prognostic scoring system and its constituent parameters.

Survival curves were calculated using the Kaplan–Meier method.

All statistical tests were 2-sided. Results were considered statistically significant when *p* < 0.05.

The tables present only variables which differ or are selected in the context of the article.

### GLVC model

According to the results of the univariate analysis, available literature data, and own clinical experience, we selected some significant variables for multivariate analysis. The significant factors identified by the subgroup step-back multivariate analysis, including hs-CRP, NTproBNP in the group of laboratory parameters; LVDD, GLPS, LVEF in the echocardiographic group; HR on admission, width of the QRS complex in the electrocardiographic group and oxygen pulse in the group of CPET parameters. After them, we selected four parameters based on literature data and our own clinical experience, including GLPS (G), LVDD (L), VO_2_/HR (V), and hs-CRP (C) to create a novel “GLVC” prognostic score. The usefulness of subsequent markers was assessed using ROC curve analysis (with the indication of the sensitivity, specificity, predictive ability).

### Study setting

This study was conducted in the Heart Failure Unit, Department of Cardiology and Congenital Heart Diseases of Adults in Polish Mother’s Memorial Hospital Research Institute, in a tertiary hospital in the city of Lodz, Poland.

The ClinicalTrials.gov database is provided herein (No 36 NCT04753814).

## Results

### Evaluation of basic characteristics of the studied groups

The median age of all patients was 64 years (range, 58–69), and 57% (65) of these patients were male. Based on the available echocardiography results (*N* = 113), the mean LVEF was 44% (IQR 39.0–50.0). Of those patients, 26% (*N* = 30) had HFpEF, 47% (*N* = 53) had HFmrEF, and 26% (*N* = 30) had HFrEF. Ischemic etiology of HF was present in 43.36% of all patients (*N* = 49). Of all patients with baseline rhythm data, 23 (20.35%) had AF, and 90 (79.65%) had SR in ECG on admission. The studied population was burdened with the most common comorbidities including arterial hypertension, coronary artery disease, diabetes mellitus, valve diseases, and hyperlipidemia. Detailed characteristics of the study population are presented in Table 2. Detailed characteristics of the subgroup population are presented in Table 3.

**Table 2:** Baseline characteristics of the study population

**Parameters**	**Values**
**Demographic characteristic**	
Age (years)	64 (58–69)
Males, *N* (%)	65 (57.52%)
**Admission parameters**	
Body mass index (kg/m^2)^	28.68 (25.64–33.27)
Body surface area (m^2^)	1.95 (1.78–2.13)
SBP (mmHg)	134 (120–146)
DBP (mmHg)	80 (70–86)
Width of the QRS complex (ms)	99 (84–121)
HR (bpm)	70 (65–80)
6MWT distance (m)	370.50 (290.70–449.20)
NYHA class	2 (2–2.5)
KCCQ score (point)	70.05 (50–83.33)
**Comorbidities**	
Arterial hypertension, *N* (%)	83 (73.45)
Coronary artery disease, *N* (%)	53 (46.90)
History of myocardial infarction, *N* (%)	37 (32.74)
Chronic kidney disease, *N* (%)	13 (11.50)
History of atrial fibrillation, *N* (%)	32 (28.32)
Diabetes mellitus, *N* (%)	31 (27.43)
COPD, *N* (%)	8 (7.1)
History of cancer, *N* (%)	19 (16.81)
History of stroke, *N* (%)	9 (7.96)
ICD, *N* (%)	4 (3.5)
CRT, *N* (%)	1 (0.9)
PCI, *N* (%)	34 (30.09)
**Echocardiography parameters**	
LVEF (%)	44 (39–50)
TAPSE (mm)	21 (18–24)
LVSD (mm)	40 (32–49)
LVDD (mm); mean ± SD	56.54 ± 8.86
LVEDV (cm^3^)	124 (91–163)
LVESV (cm^3^)	65 (45–99)
LAVI (ml/m^2^)	42.07 (34–52)
**CPET parameters**	
RER	1.08 (0.98–1.14)
VO_2_%Norm (%)	67 (57–79)
VO_2_ max (ml/kg/min)	15 (11–19)
**Laboratory tests results**	
NTproBNP on admission (pg/ml)	395 (207–935)
hs-cTnT on admission (pg/ml)	10.80 (7.70–19.40)
Hemoglobin (g/dl)	13.40 (12.50–14.50)
Sodium (mmol/l)	141 (140–142)
Potassium (mmol/l)	4.50 (4.20–4.65)
Creatinine (μmol/l)	0.88 (0.73–1.04)
CRP (mg/l)	0.50 (0.50–0.89)
Uric acid (mg/l)	6.20 (5.30–7.50)
**Selected biochemical biomarkers results**	
Neprilysin (pg/ml)	429.40 (238.50– 786.20)
Galectin-3 (ng/ml)	9.40 (6.90–12.80)
hs-CRP (µg/ml)	2.40 (0.96– 5.30)
ST-2 (pg/ml)	26.50 (15.10– 41.80)
Ferritin (ng/ml)	183.80 (53.80– 382.20)
**Medications on admission**	
ACEI, *N* (%)	75 (66.37)
ARB, *N* (%)	18 (15.93)
ARNI, *N* (%)	10 (8.85)
BB, *N* (%)	98 (86.73)
MRA, *N* (%)	52 (46.02)
Diuretics, *N* (%)	65 (57.52)
** Follow-up after discharge (12 months)**	
Rehospitalization for HF, *N* (%)	38 (33.63)
Hospitalization for cardiovascular reasons, *N* (%)	22 (19.46)
Myocardial infarction, *N* (%)	1 (0.88)
Chronic coronary syndrome, *N* (%)	16 (14.16)
Acute coronary syndrome, *N* (%)	4 (3.54)
Stroke, *N* (%)	1 (0.88)
Death from any cause, *N* (%)	5 (4.42)

Data are given as median (interquartile range) unless otherwise indicated

*SBP* systolic blood pressure, *DBP* diastolic blood pressure, *HR* heart rate, *6MWT* 6-min walk test, *NYHA* New York Heart Association, *KCCQ* The Kansas City Cardiomyopathy Questionnaire, *COPD* chronic obstructive pulmonary disease, *ICD* implantable cardioverter defibrillator, *CRT* cardiac resynchronization therapy, *PCI* percutaneous coronary intervention, *LVEF* left ventricle ejection fraction, *TAPSE* tricuspid annular plane systolic excursion, *LVSD* left ventricular systolic diameter, *LVDD* left ventricular diastolic diameter, *LVEDV* left ventricular end-diastolic volume, *LVESV* left ventricular end-systolic volume, *LAVI* left atrial volume index, *RER* respiratory exchange ratio, *VO*_*2*_*%Norm* percentiles of maximal oxygen consumption, *VO*_*2*_* max* maximal oxygen consumption, *NTproBNP* N-terminal prohormone brain natriuretic peptide, *hs-cTnT* high-sensitive cardiac troponin T, *CRP* C-reactive protein, *hs-CRP* high-sensitive C-reactive protein, *ST-2* suppression of tumourigenicity 2, *ACEI* angiotensin converting enzyme inhibitors, *ARB* angiotensin receptor blockers, *ARNI* angiotensin receptor-neprilysin inhibitor, *BB* beta blockers, *MRA* mineralocorticoid receptor antagonists

**Table 3:** Clinical characteristics and medications of patients on admission in the HFpEF, HFmrEF, and HFrEF group (the Kruskal–Wallis with Dunn post hoc test)

**Parameters**	**HFpEF**	**HFmrEF**	**HFrEF**	***p***
All patients, *N* (%)	30 (26.55)	53 (46.90)	30 (26.55)	
**Parameters on admission**				
HR (bpm)	69 (61–76)	70 (65–80)	71 (65–80)	0.13
Width of the QRS complex (ms)	85 (82–98)	100 (87–125)	114 (96–156)	**< 0.001**
QTcBAz (ms)	426 (412–437)	447.50 (431–480)	444.50 (433–484)	**< 0.001**
NYHA class				0.10
I	1 (3.33)	3 (5.66)	5 (16.67)	0.10
II	21 (70)	33 (62.26)	11 (36.67)	0.10
III	8 (26.67)	16 (30.19)	14 (46.67)	0.10
IV	0	1 (1.89)	0	0.10
Lung congestion, *N* (%)	2 (6.67)	5 (9.43)	8 (26.67)	**0.04**
Edema, *N* (%)	7 (23.33)	8 (15.09)	6 (20)	0.63
6MWT distance (m)	382.80 (271–449.20)	341.70 (289.50–422.10)	375.40 (311.60–457)	0.44
KCCQ score (point)	71.90 (59.10–83.30)	67.80 (43.20–79.40**)**	71.10 (53.10–86.50)	0.43
**HF history**				
AHF de novo, *N* (%)	10 (33.33)	6 (11.32)	3 (10)	**0.02**
Rehospitalization in the last 12 months, *N* (%)	3 (10)	20 (39.22)	15 (50)	**< 0.001**
Ischemic etiology, *N* (%)	11 (36.67)	23 (43.40)	15 (50)	0.58
Non-ischemic etiology, *N* (%)	19 (63.33)	30 (56.60)	15 (50)	0.58
**Comorbidities**				
PCI, *N* (%)	10 (33.33)	14 (26.42)	10 (33.33)	0.73
CABG, *N* (%)	1 (3.33)	3 (5.66)	3 (10.0)	0.55
ICD, *N* (%)	0	0	4 (13.33)	**< 0.001**
CRT, *N* (%)	0	0	1 (3.33)	0.25
Pacemaker, *N* (%)	0	3 (5.66)	1 (3.33)	0.41
Atrial fibrillation, *N* (%)	7 (23.33)	18 (33.96)	8 (26.67)	0.56
Coronary artery disease, *N* (%)	14 (46.67)	22 (41.51)	17 (56.67)	0.41
Mitral regurgitation, *N* (%)	12 (40)	28 (52.83)	15 (50)	0.52
Aortic regurgitation, *N* (%)	3 (10)	10 (18.87)	3 (10)	0.40
Tricuspid regurgitation, *N* (%)	12 (40)	20 (37.74)	8 (26.67)	0.50
Aortic stenosis, *N* (%)	1 (3.33)	3 (5.66)	1 (3.33)	0.84
Diabetes mellitus, *N* (%)	10 (33.33)	16 (30.19)	5 (16.67)	0.29
Arterial hypertension, *N* (%)	24 (80)	40 (75.47)	19 (63.33)	0.31
Hyperlipidemia, *N* (%)	28 (93.33)	33 (62.26)	25 (83.33)	** < 0.001**
**Laboratory test results**				
NTproBNP on admission (pg/ml)	231 (133–399)	355 (183–933)	816.0 (367–2041)	** < 0.001**
hs-cTnT on admission (pg/ml)	8.40 (5.90–11.30)	11.3 (8–22.80)	13.30 (8.80–21.10)	**0.02**
Hemoglobin (g/dl)	13(12.10–14)	13.40 (11.60–14.50)	14(13.40–15.10)	**0.02**
Sodium (mmol/l)	141 (140–142)	141 (140–143)	141.50 (140–143)	0.50
Potassium (mmol/l)	4.40 (4.20–4.60)	4.40 (4.10–4.60)	4.50 (4.30–4.80)	0.09
Creatinine (μmol/l)	0.82 (0.60–1.14)	0.88 (0.76–0.98)	0.96 (0.76–1.06)	0.35
CRP (mg/l)	0.50 (0.50–0.60)	0.50 (0.50–0.88)	0.57 (0.50–1.40)	0.11
**Selected biochemical biomarkers results**				
Neprilysin (pg/ml)	339.15 (221.82–687.58)	480.18 (323.93–792.24)	342.08 (228.75–959.99)	0.38
Galectin-3 (ng/ml)	7.96 (5.44–12.45)	9.35 (7.06–13.23)	9.98 (8.20–12.13)	0.18
hs-CRP (ug/ml)	2.24 (0.96–3.81)	2.46 (0.74–5.24)	3.09 (1.04–7.46)	0.46
ST-2 (pg/ml)	14.73 (13.10–29.45)	31.04 (19.14–47.13)	25.87 (14.73–52.45)	**0.002**
Ferritin (ng/ml)	166 (51.78–381.29)	118.18 (49.40–383.04)	232.37 (130.66–391.31)	0.31
**Medications**				
ACEI, *N* (%)	19 (63.33)	32 (60.38)	24 (80)	0.18
ARB, *N* (%)	3 (10)	11 (20.75)	4 (13.33)	0.40
ARNI, *N* (%)	0	0	10 (8.85)	** < 0.001**
BB, *N* (%)	24 (80)	45 (84.91)	29 (9667)	0.14
MRA, *N* (%)	9 (30)	23 (43.40)	20 (66.67)	**0.015**

Data are given as median (interquartile range) unless otherwise indicated

A *p* value of < 0.05 is considered statistically significant (values ​​in bold in the table)

*HR* heart rate, *QTcBaz* QT intervals reflecting cardiac repolarization were calculated by Bazett, *NYHA* New York Heart Association, *6MWT* 6-min walk test, *KCCQ* Kansas City Cardiomyopathy Questionnaire, *AHF* acute heart failure, *PCI* percutaneous coronary intervention, *CABG* coronary artery bypass graft surgery, *ICD* implantable cardioverter defibrillator, *CRT* cardiac resynchronization therapy, *NTproBNP* N-terminal prohormone of brain natriuretic peptide, *hs-cTnT* high-sensitive cardiac troponin, *CRP* C-reactive protein, *hs-CRP* high-sensitive C-reactive protein, *ST-2* suppression of tumourigenicity 2 soluble interleukin 1 receptor-like 1, *ACEI* angiotensin-converting enzyme inhibitors, *ARB* angiotensin receptor blockers, *ARNI* angiotensin receptor-neprilysin inhibitor, *BB* beta blockers, *MRA* mineralocorticoid receptor antagonists

### Association between parameters and composite primary endpoint

To determine the prognostic value of collected parameters of HF, clinical characteristics of the patients, ECG results, bioelectrical impedance body mass analysis results, echocardiography results, key CPET parameters, 6MWT results, and laboratory data, including NTproBNP, hs-cTnT, hemoglobin, sodium, potassium, creatinine, uric acid, and biomarkers including neprilysin, galectin-3, and hs-CRP, ST-2 were subjected to analyses. The univariate regression analysis (Table 4) revealed the significant variables (i.e., bolded variables with the *p*-value below 0.05) affecting composite adverse outcome occurrence in HF patients. According to the results of the univariate analysis, available literature data, and own clinical experience, we selected some significant variables for multivariate analysis. The selected multivariable analyses for each of the following areas, i.e., biochemical test, electrocardiographic, and cardio-pulmonary exercise results, were conducted. These three multivariable analyses selected the candidate markers including NTproBNP, hs-CRP, LVDD, GLPS, LVEF, and oxygen pulse for the total multivariable analysis. The results of the final multivariate analysis was presented in Table 5.

**Table 4:** Univariate analysis of the association of patients’ characteristics with the occurrence of composite endpoint

**Parameters**	**Event-free group** ***N* = 59 (52.2%)**	**Composite primary endpoint** ***N* = 54 (47.8%)**	
	**Values**		***p***
**Parameters on admission**			
DBP (mmHg)	80 (70–86)	80 (73–86)	0.33
SBP (mmHg); mean ± SD	132.84 ± 15.76	135.49 ± 17.84	0.41
HR (bpm)	70 (62–76)	75 (67–85)	**0.01**
6MWT (m)	373.15 (304.15–430.30)	340.30 (286.10–454.30)	0.39
Width of the QRS complex (ms)	94 (84–116)	105 (90–134)	**0.02**
QTcBaz (ms)	436 (422–453)	445 (431–478)	**0.04**
**Echocardiography parameters**			
LVEF (%)	47 (40–56)	41 (35–47)	**< 0.001**
LVEDV (cm3)	110.50 (87–140)	134 (112–211)	**0.01**
LVESV (cm3)	56 (36–84)	79 (56–129)	**< 0.001**
RVPs (mmHg)	28.50 (25.50–34.50)	30 (27–37)	0.35
LAVI (ml/m2)	40.50 (34–47.50)	45.90 (34.25–65.20)	0.06
TAPSE (mm); mean ± SD	21.42 ± 4.68	20.67 ± 3.60	0.34
LVDD (mm); mean ± SD	54.42 ± 8.49	58.85 ± 8.76	**0.01**
LVSD (mm)	36 (30–44)	45.50 (36–51)	**0.001**
VCI (mm); mean ± SD	16 ± 3.95	17.33 ± 4.20	0.11
GLPS (%);mean ± SD	15.29 ± 5.06	12.19 ± 4.01	**0.01**
**CPET parameters**			
RER	1.08 (0.99–1.14)	1.06 (0.97–1.14)	0.48
VO_2_ max (mL/min)	1.26 (0.89–1.69)	1.18 (0.82–1.49)	0.13
VO_2_ max/kg (mL/kg/min)	15 (12–19)	14 (10.50–18.50)	0.34
VO_2_%Norm (%); mean ± SD	71.79 ± 17.43	63.38 ± 14.54	**0.02**
VO_2_/AT; mean ± SD	0.97 ± 0.37	0.90 ± 0.26	0.29
VE/VCO_2_; mean ± SD	31.68 ± 4.86	33.81 ± 5.97	0.06
VO_2_/HR	11 (9–14)	9 (7–12.50)	**0.03**
**Laboratory tests results**			
NTproBNP (pg/ml)	263 (160–475)	638.50 (206–1606)	**< 0.001**
hs-cTnT (pg/ml)	9.60 (7.60–12.90)	16.90 (8–23.60)	**0.01**
Hemoglobin (g/dl)	13.80 (12.30–15.10)	13.35 (12.50–14.20)	0.23
Sodium (mmol/l)	141 (140–142)	141.50 (140–143)	0.33
Potassium (mmol/l)	4.40 (4.20–4.60)	4.50 (4.20–4.70)	0.25
Creatinine (umol/l)	0.83 (0.70–0.97)	0.93 (0.80–1.11)	**0.01**
Glucose (mmol/l)	91 (85–103)	93 (86–105)	0.56
CRP (mg/l)	0.50 (0.50–0.68)	0.57 (0.50–1.44)	**0.03**
Uric acid (mg/dl); mean ± SD	6.15 ± 1.48	6.65 ± 1.89	0.12
**Selected biochemical biomarkers results**			
Neprilysin (pg/ml)	404.74 (237.89–684.12)	429.51 (292.80–918.96)	0.21
Galectin-3 (ng/ml)	8.83 (6.23–13.04)	9.80 (8.07–12.79)	0.17
hs-CRP (ug/ml)	1.48 (0.79–3.85)	3.20 (1.58–8.43)	**0.01**
Ferritin (ng/ml)	205.05 (71.86–368.10)	167.18 (48.64–391.31)	0.85
ST-2 (pg/ml)	21.67 (13.68–37.07)	27.64 (18.59–47.01)	**0.03**

Data are given as median (interquartile range) unless otherwise indicated

A *p* value of < 0.05 is considered statistically significant (values ​​in bold in the table)

*DBP* diastolic blood pressure, *SBP* systolic blood pressure, *HR* heart rate, 6MWT 6-min walk test, *QTcBaz* QT intervals reflecting cardiac repolarization were calculated by Bazett, *LVEF* left ventricle ejection fraction, *LVEDV* left ventricular end-diastolic volume, *LVESV* left ventricular end-systolic volume, *RVPs* right ventricular systolic pressure, *LAVI* left atrium volume index, *TAPSE* tricuspid annular plane systolic excursion, *LVDD* left ventricular diastolic diameter, *LVSD* left ventricular systolic diameter, *VCI* vena cava inferior, *GLPS* global longitudinal peak strain, *RER* respiratory exchange ratio, *VO*_*2*_* max* maximal oxygen consumption, *VO*_*2*_*%Norm* percentiles of maximal oxygen consumption, *VO*_*2*_ oxygen uptake, *AT* anaerobic threshold, *VE* minute ventilation, *VCO*_*2*_ CO_2_ output, *VE/VCO*_*2*_ ventilatory equivalent for CO_2_, *VO*_*2*_*/HR* peak VO_2_ divided by the heart rate, *NTproBNP* N-terminal prohormone brain natriuretic peptide, *hs-cTnT* high-sensitive cardiac troponin T, *CRP* C-reactive protein, *hs-CRP* high-sensitive C-reactive protein, *ST-2* suppression of tumourigenicity 2

**Table 5:** Final multivariate analysis of the association of patients’ characteristics with the occurrence of composite endpoint

Final multivariate analysis				
Variables	Exp(B)–OR	95% CI for OR		*p*
LVDD (mm)	4.84	1.79	13.05	**0.002**
VO_2_/HR	4.26	1.55	11.69	**0.004**

Data are given as median (interquartile range) unless otherwise indicated

A *p* value of < 0.05 is considered statistically significant (values ​​in bold in the table)

*DBP* diastolic blood pressure *SBP* systolic blood pressure, *HR* heart rate, 6MWT 6-min walk test, *QTcBaz* QT intervals reflecting cardiac repolarization were calculated by Bazett, *LVEF* left ventricle ejection fraction, *LVEDV* left ventricular end-diastolic volume, *LVESV* left ventricular end-systolic volume, *RVPs* right ventricular systolic pressure, *LAVI* left atrium volume index, *TAPSE* tricuspid annular plane systolic excursion, *LVDD* left ventricular diastolic diameter, *LVSD* left ventricular systolic diameter, *VCI* vena cava inferior, *GLPS* global longitudinal peak strain, *RER* respiratory exchange ratio, *VO*_*2*_* max* maximal oxygen consumption, *VO*_*2*_*%Norm* percentiles of maximal oxygen consumption, *VO*_*2*_ oxygen uptake, *AT* anaerobic threshold, *VE* minute ventilation, *VCO*_*2*_ CO_2_ output, *VE/VCO*_*2*_ ventilatory equivalent for CO_2_, *VO*_*2*_*/HR* peak VO_2_ divided by the heart rate, *NTproBNP* N-terminal prohormone brain natriuretic peptide, *hs-cTnT* high-sensitive cardiac troponin T, *CRP* C-reactive protein, *hs-CRP* high-sensitive C-reactive protein, *ST-2* suppression of tumourigenicity 2

### Personalized scoring system to predict composite adverse outcomes

Based on literature data and our own clinical experience, the four following markers, i.e., GLPS (G), LVDD (L), VO_2_/HR (V), and hs-CRP (C), were used to create the personalized scoring system (GLVC). All of these selected parameters (G, L, V, C) have a significant influence on the adverse outcome, i.e., re-hospitalization or sudden death.

**Fig. 1:**
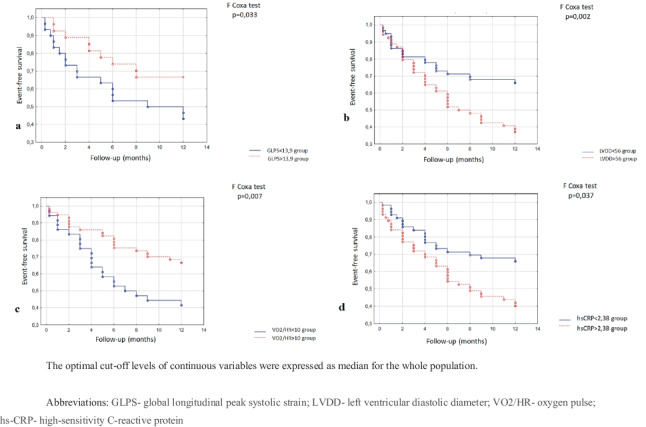
The Kaplan–Meier survival curves estimated on composite adverse outcome. **a** GLPS ≤ − 13.9% (blue line) or GLPS > − 13.9% (red line). **b** LVDD ≤ 56 mm (blue line) or LVDD > 56 mm (red line). **c** VO2/HR ≤ 10 (blue line) or VO2/HR > 1 (red line). **d** hs-CRP ≤ 2.38 µg/ml (blue line) or hs-CRP > 2.38 ug/ml (red line)

The Kaplan–Meier curves for the estimated composite adverse outcomes are presented based on hs-CRP, LVDD, GLPS, and oxygen pulse in Fig. 1.

**Fig. 2:**
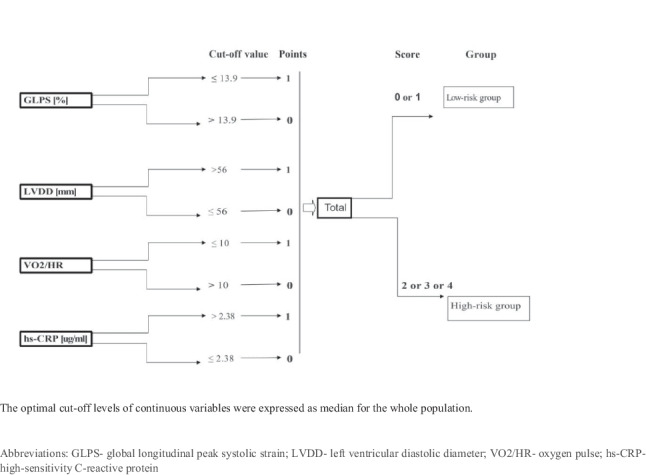
Calculation of the GLVC prognostic score

The GLVC score system was calculated according to the formula (Fig. 2): 1 point for low GLPS (≤ − 13.9%), high LVDD (> 56 mm), low oxygen pulse (≤ 10), and high hs-CRP (> 2.38 µg/ml), and 0 points for high GLPS (> − 13.9%) low LVDD (≤ 56 mm), high oxygen pulse (> 10), low hs-CRP (≤ 2.38 µg/ml). The total score is the sum of four factors and ranges from 0 to 4. To optimize the scoring system, we defined scores of 0 and 1 as the low-risk group (*N* = 57, 50.4%) and scores of 2, 3, and 4 as the high-risk group (*N* = 56, 49.6%) (Fig. 2).

On the basis of the optimal cut-off values (median of the continuous variables), we evaluated the diagnostic performance of the GLPS, LVDD, VO_2_/HR, hs-CRP, and GLVC by using ROC curve analysis.

**Fig. 3:**
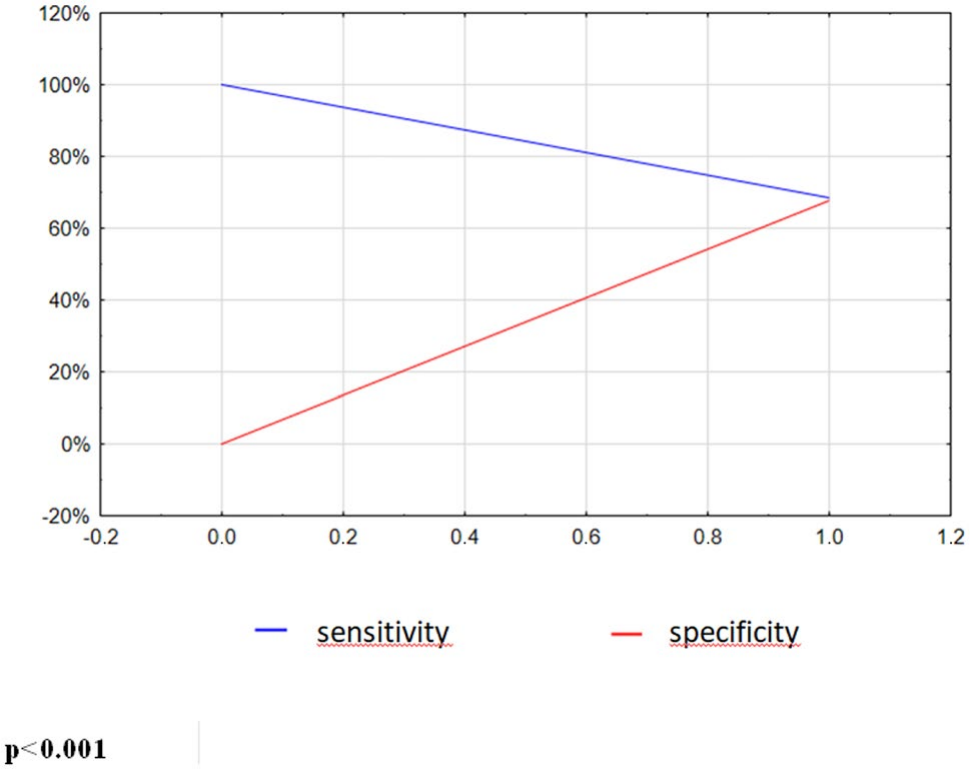
Sensitivity (blue line) and specificity (red line) of GLVC

**Fig. 4:**
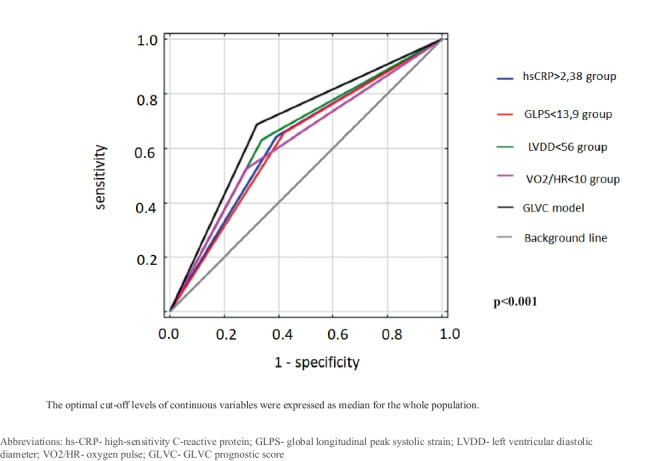
Receiver operating characteristic (ROC) curve analyses for GLVC prognostic score and its constituent parameters

The area under the curve, sensitivity, and specificity of GLVC and its constituent parameters are shown in Table 6 and Fig. 3. The receiver operating characteristic (ROC) curve for GLVC and its constituent parameters are presented in Fig. 4.

**Table 6:** Cut-off values and AUC for GLVC prognostic score and its constituent parameters

**Prognostic system**	**Cut-off value**	**AUC**	**Sen. (%)**	**Spec. (%)**	***p***
GLPS (%)	13.9	0.617	65.4	58.1	0.119
LVDD (mm)	56	0.645	63.0	66.1	< 0.001
VO_2_/HR	10	0.621	52.5	71.7	0.042
hs-CRP (µg/ml)	2.38	0.626	64.2	61.0	0.018
GLVC	-	0.682	68.5	67.8	< 0.001

The optimal cut-off levels of continuous variables were expressed as median for the whole population

*GLPS* global longitudinal peak systolic strain, *LVDD* left ventricular diastolic diameter, *VO*_*2*_*/HR* oxygen pulse, *hs-CRP* high-sensitivity C-reactive protein, *GLVC* GLVC prognostic score, *AUC* area under the curve, *Sen.* Sensitivity, *Spec.* specificity

**Fig. 5:**
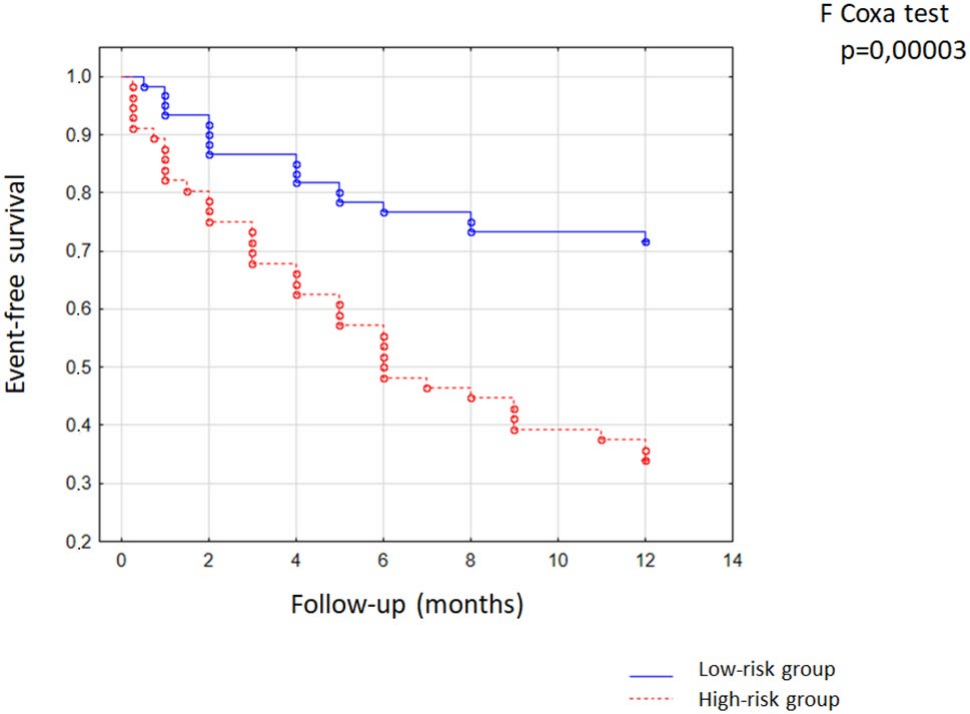
The Kaplan–Meier survival curves estimated on the adverse outcome of the whole cohort according to the GLVC prognostic score

The Kaplan–Meier analyses and log-rank tests were performed to determine the prognostic value of GLVC in predicting adverse outcomes in patients with HF. The data demonstrated that adverse outcomes in the high-risk group were significantly higher than those of the low-risk group (log-rank test: *p* < 0.001, Fig. 5).

## Discussion

Our study is the first one which collected data coming from a single cardiology department and built-up a novel, independent of the ejection fraction, score for HF patients in order to predict the adverse outcome and to precisely select a group of patients that in the first place needs close monitoring of the health condition and maximum optimization of therapy.

Over the years, many predictors for HF exacerbation and mortality have been identified, including clinical, laboratory, and echocardiographic parameters. Several HF risk scores are currently used in clinical practice, for example, the Meta-Analysis Global Group in Chronic Heart Failure (MAGGIC-HF) risk score [20] in HFrEF and HFpEF population and Seattle Heart Failure Model (SHFM) [21] in HFrEF patients. Despite the many risk models developed for patients with HF, there are still no comprehensive prognostic data, and it is the subject of ongoing research. The research carried out as part of this study identified independent variables associated with poor prognosis in a 1-year follow-up of patients hospitalized for HF. We made easy and useful scoring based on biomarker of inflammation, i.e., high-sensitivity–reactive protein (hs-CRP), impaired left ventricular (LV) relaxation assessed as higher left ventricular diastolic diameter (LVDD), early predictor of LV systolic dysfunction, i.e., global longitudinal peak strain (GLPS) and a measure of the metabolic efficiency of the heart muscle, i.e., oxygen pulse (VO_2_/HR) in HF patients.

The role of GLPS as a marker of cardiovascular events in HF population has been studied in some important publications over the years. In the Cho et al. study, GLPS was a predictor of the composite endpoint (cardiac mortality and HF exacerbations) in acute HF patients [22]. Nahum et al. has proved useful of GLPS in the patients with chronic HF with diverse etiology [23]. Buggey et al. demonstrated the relationship between GLPS and composite endpoint (mortality or hospitalization for HF exacerbation) in patients with acute HF and preserved ejection fraction (≥ 50%) [24]. In Kaufmann et al. study, GLPS was a useful tool to predict heart failure exacerbation in stable outpatients with ischemic left ventricular systolic dysfunction [25]. Global longitudinal strain is known as a parameter which is more sensitive and objective than LVEF in the evaluation of LV abnormalities in cardiovascular diseases [26, 27]. GLPS cut-off value of -13.9% evaluated in the present study is within the range of values ​​ presented by other authors, varying from about -7 to -14% [25, 28–30]. The value of this parameter is usually better in groups of patients with higher LVEF and worse in those with lower LVEF.

Although the role of EF in heart failure has been studied over the years, the LV diastolic diameter obtained routinely along with LVEF has not been considered for potential use in risk stratification yet. Left ventricle diastolic size could be a more stable measure in clinical practice. A potential limitation of EF is significant subjectivity. Publications have suggested that such variability in EF could be in the range of 10% or greater [31]. Moreover, Lee et al. determined that LV dilatation was an independent predictor of overall and sudden death in the HF patients group [32]. Another, Watanabe et al. analysis in a congestive heart failure population revealed that patients with sudden death had a higher mean LV diastolic diameter [33]. Also, Narayanan et al. suggested LV diameter as a risk predictor for SCD independent of the LVEF in chronic heart failure patients. Categorization of HF patients using LV diameter may provide a practical tool for risk assessment in this population of the patients. LVDD cut-off value of 56 mm evaluated in the present study is within the range of values ​​ presented by other authors, varying from about 52 to 59 mm [32–34].

Oxygen pulse (VO_2_/HR) is a measurement of peak VO_2_ corrected for heart rate. It is a noninvasive indicator of stroke volume and arterio-venous oxygen difference. This parameter could be more dependent on cardiac pump function reserve because traditional measures of peak VO_2_ ignore heart rate response during exercise. This correlation was observed and explored earlier in some other studies. Cohen-Solal et al. study [35] revealed a nonsignificant trend for higher peak O2 pulse in 143 survivors than in 35 patients who died (8.8 vs 8.4 ml/beat; *p* = 0.1). In another study [36], oxygen pulse has been proven to be higher in 115 event-free patients with HF than in 32 patients who underwent transplantation (11.4 vs 9.2 ml/beat, *p* = 0.05), with a strong trend toward lower values in those who died (mean 9.8 ml/beat). Also, Lavie et al. [37] demonstrated that the peak O2 pulse was significantly higher in event-free subjects than in the group with clinical events. The oxygen pulse cut-off value of 10 ml/beat separated those HF patients with clinical events from event-free subjects evaluated in the present study is similar to values ​​ presented by other authors [35–37].

Hs-CRP cut-off value of 2.38 ng/l separated those HF patients with clinical events from event-free subjects evaluated in the present study conforms to values ​ presented by other authors. Moliner et al. revealed that more than half of hospitalized HF patients had a hs-CRP ≥ 2 ng/l [38]. Also in Pellicori et al. study [39], about 70% of patients with HF had a hs-CRP ≥ 2 mg/l (median of plasma hs-CRP for patients diagnosed with HF was 3.9 mg/l (IQR 1.6–8.5)), which was associated with a lower LVEF and fluid congestion. Moreover raised plasma concentrations of hs-CRP were useful to predict a higher all-cause mortality rate, independent of age, HF symptoms, renal function, and NT-proBNP value. Increased hs-CRP is correlated with increases in mortality due to cardiovascular (CV) and non-CV causes (for all-cause mortality HR 2.49 (95% CI 2.19–2.84; *p* < 0.001); for CV mortality HR 2.26 (95% CI 1.91–2.68; *p* < 0.001), and for non-CV mortality HR 2.96 (95% CI 2.40–3.65; *p* < 0.001)) [39]. Also, another data from randomized controlled trials have shown that high levels of hs-CRP are associated with adverse CV outcomes in patients with HFrEF, and there are similar findings from smaller studies enrolling HFrEF or HFpEF patients [40–42].

By including all four biomarkers in the GLVC scoring system, we covered impaired LV diastolic and systolic function, impaired metabolic efficiency of the heart muscle, and other aspects such as inflammation in HF population.

The big advantage of our model is being a comprehensive evaluation system that represents a patient’s condition from multidimensional HF aspects. However, all data in this study were collected from one relatively small sample of patients hospitalized due to HF. Therefore, large, multicenter studies are needed in the future.

### Study limitations and strengths

The current study has some limitations. The first and critical limitation is that there are no different datasets for a derivation and a validation. This is a preliminary study based on the small study sample from a single center. Second, the patient population was limited to those hospitalized due to HF in our Department, which could evoke referral bias because patients referred for hospitalization are not representative of the general HF population. The latest European Society of Cardiology guideline for HF [8] categorizes EF into 3 groups: HFrEF (EF < = 40%), HFmrEF (EF = 41–49%), and HFpEF (EF > = 50%). We could not analyze our model according to this classification because of the small sample size. Third, because the disease severity in our patients was mild or moderate and the study population included only Polish patients, the results should be carefully interpreted when applied to different populations. However, our GLVC scoring predicting system confirmed efficiency in risk assessment of HF patients independently from left ventricular ejection fraction as well as in predicting event-free survival in this group of patients. This is a personalized easy, quick, and cheap tool which may help clinicians to select the HF patients which need at first the close monitoring and optimalization of treatment according to current guidelines. Taking into account a huge population of patients with HF, this kind of personalized tool that gives a comprehensive evaluation of a multidimensional HF system might be useful in everyday practice.

## Conclusion

The novel personalized scoring system, based on biomarker of inflammation, i.e., high-sensitivity–reactive protein (hs-CRP), impaired left ventricular (LV) relaxation assessed as higher left ventricular diastolic diameter (LVDD), early predictor of LV systolic dysfunction, i.e., global longitudinal peak strain (GLPS) and a measure of the metabolic efficiency of the heart muscle, i.e., oxygen pulse (VO_2_/HR), in HF patients, is an easily available and effective tool and could be taken into account in the assessment prognosis of HF patients in the future. A personalized diagnostic process using the ECHO, CPET, and inflammatory biomarkers to assess the hemodynamic and clinical condition of individual patients may help identify those with a poorer prognosis.

## Data Availability

Data was collected and processed maintaining the confidentiality of the patients and physicians participating in the study.
